# SUSTAINABLE DEMENTIA CARE: CLINICAL, PROGRAMMATIC, AND PUBLIC HEALTH PERSPECTIVES

**DOI:** 10.1093/geroni/igae098.0255

**Published:** 2024-12-31

**Authors:** Joseph Gaugler, Elma Johnson, Laura Gitlin

**Affiliations:** Chair; Co-Chair; Discussant

## Abstract

When considering the dissemination and implementation of evidence-based innovations in aging, sustainability is a driving concern but understudied outcome. Sustainability refers to “is the extent to which a newly implemented intervention is maintained or institutionalized within a service setting’s ongoing, stable operations” (Lewis et al., 2017). Although attention in dementia care is beginning to pivot to issues related to successful dissemination and implementation of a growing number of evidence-based innovations, concerns related to the maintenance of long-term care adoption of dementia care interventions in clinical, community, and public health contexts remain pressing. Beyond dementia care and in the broader field of implementation science, available research on sustainability is limited with challenges ranging from heterogeneity in definitions, measurement, follow-up periods and similar issues. The objective of this symposium is to initiate a scientific and practice agenda as it relates to sustainability for dementia care interventions. We will feature presentations on dementia care sustainability from public health, community, clinical, and scientific perspectives. Following this symposium, we aim to establish a framework to better achieve sustainability of evidence-based and evidence-informed dementia care innovations across key settings and communities.

